# Nurse’s Role from Medical Students’ Perspective during Their Interprofessional Clinical Practice: Evidence from Lithuania

**DOI:** 10.3390/healthcare9080963

**Published:** 2021-07-29

**Authors:** Aurelija Blaževičienė, Aurika Vanckavičienė, Renata Paukštaitiene, Asta Baranauskaitė

**Affiliations:** 1 Nursing and Care Department, Lithuanian University of Health Sciences, 50161 Kaunas, Lithuania; aurika.vanckaviciene@lsmuni.lt; 2 Department of Physics, Mathematics and Biophysics, Lithuanian University of Health Sciences, 50161 Kaunas, Lithuania; renata.paukstaitiene@lsmuni.lt; 3 Rheumatology Department, Lithuanian University of Health Sciences, 50161 Kaunas, Lithuania; asta.baranauskaite@lsmuni.lt

**Keywords:** interprofessional education, medical students, nurse role, clinical practice

## Abstract

*Background:* Attitudes towards interprofessional education are key factors that shape students’ behaviour during interprofessional practice. An interprofessional approach to training and practice is “unique”, important, and challenging. Interprofessional education allows for a deeper understanding and analysis of problems from perspectives different to those of “us”. The aim of the study was to assess medical students’ attitudes toward the nurse’s role during their interprofessional clinical practice. *Methods:* This study used a descriptive, correlational design. *Results:* Lithuanian medical students were statistically significantly more likely to think that the role of a nurse was clear and transparent to other professionals and that nurses exuded a high degree of professionalism, sought a high degree of involvement with the patient, and built deep relationships with the patients. Foreign medical students were statistically significantly more likely to believe that nurses worked more effectively alone than in a team and that they worked with the patients within their own professional field of knowledge rather than referring patients to other professionals. *Conclusions:* After 6 months of interprofessional training with nurses in the hospital, medical students gain a more clear professional picture of the role of the nurse.

## 1. Introduction

Perceptions and attitudes towards interprofessional education (IPE) are key factors that shape students’ behaviour during interprofessional practice [1]. Study programs for healthcare professionals must be designed to enable learners to acquire the interprofessional knowledge required to work in a multidisciplinary team [2].

It is also important to support the development of relationships with other professionals during the learning process [3]. Meeting new people from different professional backgrounds is clearly valued and felt to be beneficial to preparation for clinical practice [4]. However, the cultural and social environment, attitudes towards democracy, individualism, traditions, and level of development of a country are important factors that may explain how and why IPE has evolved differently across countries [5].

This qualitative study revealed that teachers’ knowledge and skills in IPE were among the main prerequisites for a positive experience in teaching and learning in IPE. The study participants emphasized that it was essential that the teacher had knowledge, skills, and an enthusiastic attitude that would motivate students and engage them in IPE [6].

In the IPE of medical students with nurses during their clinical practice, the goal is for the students to learn and perform the functions of a nurse; consequently, it is important to clarify their understanding of the nurse’s role [7]. For medical students, performing the functions of nurses during interprofessional practice is a transformative way to learn interprofessional competencies [8].

Interprofessional collaboration is an integral component of healthcare and is associated with better health outcomes for patients [7]. Members of interprofessional teams are more satisfied with their work and wellbeing. In recognition of this, the World Health Organization published a framework for action on interprofessional education (IPE) in which it outlined supporting evidence and strategies for implementing IPE in various healthcare disciplines and implored educators to implement these programs to develop a collaborative, practice-ready workforce [9].

Studies have shown that students in medical and nursing programs have poor interactions with each other during clinical practice [10]. There is a knowledge gap concerning the roles and responsibilities of nurses and how to work together effectively.

Raurell-Torredà M et al., in a randomized clinical trial, showed that using the curriculum simulation/role-play teaching methods increased the awareness of students and other team members’ roles, gave them greater confidence in their patient assessments, implemented optimal patient interventions, and showing an enhanced capacity to share key information with team members. In sum, simulations in a university setting enable trainee nurses to develop the teamwork and communication skills that they will need in their future careers [11].

Experience suggests that medical staff begin their in-hospital rotations with limited knowledge about the wide variety of healthcare professionals and the critical roles that they perform in patient care [10,12].

Little is known about the ways in which nurses and medical students learn and understand the roles and responsibilities of each profession in an interprofessional team, although nurses have a high degree of interdisciplinary collaboration [13], and when working in a multidisciplinary team, they focus on collaboration, which is a key strategy to improve services for patients [14].

When evaluating nursing practice during crises, particularly those that place the nurse in mortal danger, it is important to acknowledge both the physical and emotional impacts [15]. During COVID-19, students’ experiences during clinical practice and their attitudes towards the nursing profession and understanding of the nurse’s role are very important and may be different from those under “normal” conditions. Thus, it was particularly interesting and unique to understand fourth-year medical students’ attitudes towards the nurse’s role and their perception of interprofessional collaborative practice (IPCP) during the COVID-19 pandemic period.

The study aimed to assess medical students’ attitudes toward the nurse’s role during their interprofessional clinical practice.

## 2. Materials and Methods

### 2.1. Study Design

This study used a descriptive, correlational design.

### 2.2. Medical Programme Description

The duration of the medical program is 6 years (360 ECTS credits). Both Lithuanian students (who study in their native language) and foreign students (who study in English) participate in this program. Interprofessional training and familiarization with the functions of future team members in the healthcare system begins during the first year of study. In the first and third years of study, students in the medical program learn basic preclinical skills from a nurse instructor. In addition, third-year students studying in Lithuanian study interprofessional communication with students in the nursing program; however, students who study in the English language do not have this course. Clinical skills before clinical training are taught in the Medical Simulation Center using the HybLab teaching method, which allows for the acquisition of clinical skills in a safe environment.

**Figure 1:**
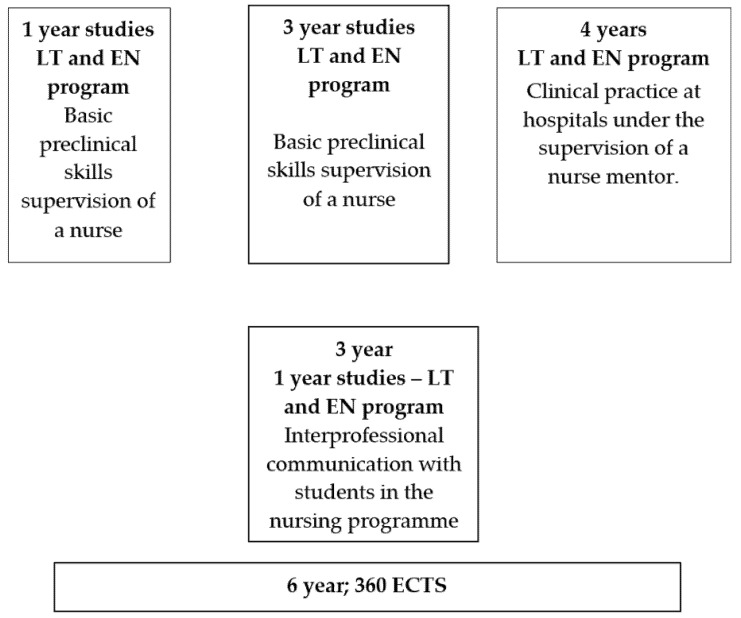
Medical program curriculum structure.

In the fourth year of the study, one day per week throughout the semester, medical students undergo clinical practice, performance of procedures, and the practice of effective communication (9 ECTS credits) at hospitals under the supervision of a nurse mentor (Figure 1).

### 2.3. Participants

The study involved 236 fourth-year students in the medical program who were studying in Lithuanian and in English (120 students studying in Lithuanian and 106 students studying in English). From February to August, the students underwent interprofessional practice on an individual schedule because of limitations related to COVID-19. Inclusion criteria were as follows: successful completion of the mandatory third-year modules and do not have academic dept. Excluded were students who had not received the entirety of their nursing training at the university level.

The students completed a questionnaire-based survey in August 2020 on the last day of their clinical practice in hospitals. The students’ clinical practice in hospitals took place under partial or strict quarantine conditions. In total, 229 questionnaires were completed correctly and were used for further analysis (the response rate was 97%).

### 2.4. Settings

The students were placed in a municipal hospital that provides a wide range of healthcare services. They worked in interprofessional teams consisting of one or two medical students and a nurse mentor. The interprofessional placement enabled students to interact with health professionals to support the care of patients with various diseases.

A nurse mentor and a nurse educator took the overall responsibility for facilitating the placement and jointly conducted teaching activities based on cases. The students participated in collaborative patient reviews that included nursing procedures and physical assessments, and they assisted in the delivery of outpatient care under the supervision of physicians and nurses.

### 2.5. Questionnaire Instrument

To assess students’ perceptions of nurses’ work and its importance, we used the role perception questionnaire. The role perception questionnaire (RPQ) was developed to evaluate undergraduate medical students’ perceptions about the roles of other professions in interprofessional education [16]. The RPQ can be used to measure changes in the role perceptions of a range of professions. The role perception questionnaire is a 20-item tool with a 10-point scale used to obtain the respondent’s views of the role of another profession and determine the extent to which the targeted professions’ role requires them to collaborate with others. Respondents are required to circle the point on the scale that represents their view.

The pilot test was carried out in one group of Lithuanian medical program students (N-15) and one group of English language program students (N-15). Two statement were excluded (Has a caring role and people skills vs. Has a technical role and Has a high opinion of their own profession vs. values their own and other professions) because more than half of the respondents during the pilot study did not mark them and we realized that they are not appropriate statements for them.

Therefore, in the final analysis, we used 18 questions. The Cronbach’s alpha coefficient of the questions is equal to 0.89.

### 2.6. Statistical Analysis

Factor analysis was applied. The Kaiser–Meyer–Olkin (KMO) value of the questionnaire was 0.865, and the value of Bartlett’s test of sphericity was *p* < 0.001, which indicates that the variables were not independent and were well suited for factor analysis. Principal component analysis and orthogonal varimax rotation were used to identify the factors. A question was assigned to the factor in which its weight was the greatest. We identified four factors that generally explained 58.58% of the original data variance. In addition, the Mann–Whitney test was applied, and medians and quartiles were calculated.

## 3. Results

### 3.1. Demographic Characteristics

The study involved fourth-year students in the medical program. Of these, 106 were foreign students and 120 students were Lithuanian. By gender, 63% (*n* = 143) of the students were women and 37% (*n* = 83) were men.

### 3.2. Medical Students’ Attitudes of the Nurse’s Role

The Mann–Whitney test (a nonparametric test) showed that Lithuanian medical students were statistically significantly more likely to think that the role of nurses was clear and transparent to other professionals and that nurses exuded a high degree of professionalism, sought a high degree of involvement with the patient, and built deep relationships with patients. In addition, Lithuanian medical students stated that, in their work, nurses engaged in a considerable amount of collaborated with others. In comparison, foreign medical students were statistically significantly more likely to believe that nurses worked more effectively alone than in a team and that they worked with the patients within their own professional field of knowledge rather than referring patients to other professionals (Table 1).

In the factor analysis, four factors were identified: breadth of professional outlook; projected professional image; possesses skills for a wide professional scope; and degree of professional interdependence. Together, these domains explained 58.58% of the original data variance. Table 2 provides information on the identified factors—i.e., the questions and their weights on the factors after varimax rotation. As the presented data show, in the first factor, breadth of professional outlook, “communicates with many professionals vs. communicates with few other professionals” had the greatest weight. Analysis of another factor, projected professional image, and its components showed that the most important components were “the role is clear and transparent to other professionals vs. uncertainty among other professionals about what the role involves” and “has a broad range of life experience vs. has little practical life experience”. The most important component of the next factor, possesses skills for a wide professional scope, was “demonstrates a sense of humour when undertaking his or her role vs. demonstrates a serious attitude when undertaking his or her role”. Finally, for the last factor, degree of professional interdependence, the component “has a specific role that involves little collaboration with others vs. engages in considerable collaboration with others” had the greatest weight (Table 2).

The analysis of the importance of the factors that define the role of the nurse and the content of his or her work from the point of view of Lithuanian and foreign medical students revealed statistically significant differences. The weights of the factor scores in the group of foreign medical students were higher; thus, we conclude that these students evaluated the nursing profession more critically (Table 3).

## 4. Discussion

### 4.1. Breadth of Professional Outlook

The Institute of Medicine (IOM, 2010) asserts that nurses are key implementers of healthcare reform and need to provide quality, safe, and patient-centred services, which results in constant change in the roles of nurses and increasing multitasking in their activities. It is thus essential that nurses themselves, as well as other health professionals and the general public, have a clear understanding of the functions and roles of nurses.

At present, nurses are adopting new roles, including coordinating care from multiple providers, managing caseloads of patients with intense care needs, and helping patients transition out of hospitals and into the home or other settings. They are working as “health coaches” and in other ways to prevent illness and promote wellness. They are also charting new paths in emerging fields such as telehealth, informatics, genetics, and genomics and are working as scientists and leaders in society [17].

The functions of a nurse include patient education and health promotion. Our research data revealed that, after 6 months of practice, the medical students (both Lithuanian and foreign) stated that nurses were involved in patients’ health education and that they were competent and capable of working with a wide range of patients. However, the medical students also stated that nurses’ communication with patients was superficial. A study by Iranian researchers on the professional communication of nurses with patients confirms our results: in that study, 80% of the patients were dissatisfied with their communication with nurses, stating that the nurses communicated superficially and those patients did not even know who their nurse was [17]. Nonetheless, professional communication between nurses and patients is one of the most important factors determining patient satisfaction with nursing care.

Teamwork is inevitable in the healthcare system and allows the provision of patient-centred care. Therefore, it is very important that team members have a clear understanding of the other professions with which they work. Effective collaboration in the healthcare environment requires intentional knowledge sharing and shared accountability for patient care [18]. Our study revealed that students in the medical program who were studying in English were statistically significantly more likely than those studying in Lithuanian to state that the nurses with whom they interacted worked more effectively alone than in a team.

### 4.2. Professional Image of Nurse and Professional Interdependence

The image of nurses has always been a matter of concern for both representatives of the nursing profession itself and those in other professions. Traditional registered nurses are adopting expanded roles as the healthcare system evolves to meet new needs. Once viewed as subservient and subordinate, nurses are now serving as full and essential partners in interdisciplinary healthcare teams [19]. However, there is little research analysing how the role of the nurse is perceived by other team members. In addition, research has shown that students in health sciences program have traditionally had very limited opportunities to interact with one another, which creates certain barriers to working in multidisciplinary teams [20].

Despite the changing roles and responsibilities of nurses in the healthcare system, the traditional perception of the role of a nurse is still widespread. This has been confirmed in a study by Norwegian researchers that revealed that, among medical and nursing students, stereotypes still have a significant impact on professional attitudes towards taking and sharing responsibilities and on perceptions of professional autonomy, which hinders teamwork in clinical practice [21,22]. However, other authors indicate that many physicians report that they have learned a great deal from nurses by working with them [23], and observational research provides evidence that nurses contribute significantly to the interprofessional education of students in clinical settings [24]. However, some research suggests that nurses and medical students lack clarity about learning roles and learning goals [25,26].

Our data revealed that medical students who were studying in Lithuanian had a clearer understanding of the nursing profession and nurses’ professional image than foreign medical students who were studying in English. The students who were studying in Lithuanian stated that the role of a nurse was clear and transparent to other professionals and that nurses exuded a high degree of professionalism, considered their professional image, and cared for their patients’ wellbeing. We can assume that the better understanding of the nursing profession and nurses’ image among the students at the Faculty of Medicine who were studying in Lithuanian occurred because they participated in an interprofessional communication course along with students in the nursing program. In addition, there was no language barrier between the medical students and nurses. In contrast, the medical students who were studying in English did not have that course in their study program, and the language barrier may have affected their communication with nurses and patients. Participation in interprofessional training can improve students’ understanding of the identity of a profession [27]. This has been confirmed by a study conducted by U.K. researchers in which medicine and nursing students who participated in a sim-IPE session had a clearer and better understanding of each other’s professions and greater teamwork abilities after the session compared with students who did not take part in that session [28].

Some researchers have commented on similar vulnerabilities and challenges of transdisciplinary practices including lack of clarity of problem definition, unbalanced problem ownership, conflicting methodological standards, and outcomes or solutions that have limited legitimacy and case-specificity [29,30].

Interprofessional training is always much better understood when it takes place in a real clinical environment rather than in classrooms because the real-life setting allows students to become immersed in the context and understand the importance and contribution of other professions [30]. An authentic context has a significant impact on the ways students perceive their learning experiences. Nevertheless, there are various challenges for sustainable implementation of IPE, including (a) non-coordinated and strictly separate curricula of different health care professions, (b) an insufficient number of specifically qualified teaching staff, and (c) limited financial resources of the institutions [8,13,19,20].

The study revealed gaps in role perception. Students in the nursing program had a more accurate perception of the physician’s role than medical students had of the nurse’s role. More accurate role perception was associated with a more positive attitude towards collaborative physician–nurse decision making [31]. A lack of participation in team duties, which medical students described as “nursing work”, was a barrier to IPE.

The medical students (both those studying in Lithuanian and those studying in English) who participated in our study stated that nurses were not independent and that their practice needed to be directed or supervised by other professionals (5.90 and 6.15 out of 10 possible points, respectively). This shows that, in Eastern European countries, despite the progress of nursing science and practice, nurses are often still positioned as “assistants” rather than as independent specialists. On the other hand, physicians also often fail to allow nurses to be equal members of the team, and this sort of presentation of the nurse’s role in the hospital distorts the desired reality for future physicians.

## 5. Conclusions

After 6 months of interprofessional training with nurses in the hospital, medical students gain a more clear professional picture of the role of the nurse. However, foreign medical program students emphasise that nurses worked more effectively alone than in a team, used only technical skills, and communicated superficially with patients. Therefore, foreign medical students have more critical attitudes about the nurse’s role owing to the language barrier and culture differences.

### 5.1. Implication for Education and Practice

Interprofessional training from the first year of studies is necessary for medical students to achieve an improved understanding of professional roles and responsibilities, effective communication, and effective teamwork.

### 5.2. List of Abbreviations

IPE—interprofessional education.

### 5.3. Limitations

Despite the fact that this study is one of the first to analyse medical students’ perceptions of the profession of a nurse, the future team member, and the limits of competence and the responsibility of the representatives of this profession, it has some limitations.

We believe that one of them is the cultural differences that may have affected the perception of a nurse’s profession among Lithuanian and foreign students. In addition, the language barrier between foreign students and nurses and patients may have led to those students’ more critical approach to the profession of a nurse.

Another limitation is that, because clinical practice took place during the COVID-19 pandemic period, epidemiological constraints may have reduced the ability of team members to communicate and may have prevented full involvement of medical students in clinical practice.

Therefore, the elimination of two statements could not allow drawing the full picture of a nurse’s role in healthcare team from the medical student’s perspective.

## Figures and Tables

**Table 1:** Medical students’ attitudes towards the work of nurses.

Item		Mean Score	LT	EN	*p*
			Median (Lower Quartile, Upper Median Quartile		
1	Role is clear and transparent to other professionals vs. Uncertainty among other professionals about what the role involves	2.98	2 (1–4)	4 (2–6)	<0.001
2	Exudes a high degree of professionalism vs. Does not appear to consider his or her professional image	3.16	2 (1–4)	3 (2–5)	0.015
3	Has a broad range of life experience vs. has little practical life experience	3.19	2 (2–5)	3 (3–5)	<0.05
4	Seeks a high degree of involvement with patients vs. Maintains a low degree of involvement with patients	3.38	3 (1.25–5)	5 (3–6)	<0.001
5	Has a health education role vs. Role is unrelated to health education	3.57	3 (1.75–5)	4 (2–5)	0.137
6	Able to work with a wide spectrum of patient/client types vs. Able to work with only a narrow range of patient/client types	3.90	3 (2–5)	4 (2.5–6)	0.039
7	Communicates with many professionals vs. Communicates with few other professionals	4.15	3 (2–6)	5 (2–6)	0.089
8	Cares for the patient’s general wellbeing vs. Cares for the patient only in relation to his or her specific professional context	4.43	5 (3–6)	5 (3–6)	0.166
9	Builds a deep relationship with the patient vs. Has a more superficial relationship with the patient	4.49	3 (2–5)	5 (4–7)	<0.001
10	Works effectively in a team vs. Works more effectively alone	4.50	4 (2–6)	5 (3–7)	0.002
11	Has an objective, medical perspective vs. Has a subjective, social perspective	4.63	5 (3–6)	5 (4–6)	0.289
12	Requires a high level of technical skill vs. Requires a high level of intellectual skill	4.72	5 (3–5)	5 (3–6)	0.123
13	Has the ability to refer a patient to another professional vs. Works with the patient within his or her own professional field of knowledge	4.90	4 (2–6)	6 (4–8)	<0.001
14	Possesses good interpersonal skills with an individual patient vs. Demonstrates good interpersonal skills within a group situation	5.08	5 (4–6)	5 (4–7)	0.167
15	Medical focus of the work vs. Social focus of the work	5.28	5 (4–6)	5 (4–6)	0.72
16	Demonstrates a sense of humour when undertaking his or her role vs. Demonstrates a serious attitude when undertaking his or her role	5.55	5 (4–7)	5 (4–7)	0.76
17	Works autonomously vs. Works with direction or supervision by another professional	6.02	6 (5–8)	6 (5–8)	0.452
18	Has a specific role that involves little collaboration with others vs. Engages in considerable collaboration with others	8.53	10 (9–10)	8 (6–9)	<0.001

**Table 2:** Factor analysis of medical students’ perceptions of the role of nurses.

Item		Breadth of Professional Outlook	Projected Professional Image	Possesses Skills for a Wide Professional Scope	Degree of Professional Interdependence
		Factor 1	Factor 2	Factor 3	Factor 4
1	Communicates with many professionals vs. Communicates with few other professionals	0.791			
2	Builds a deep relationship with the patient vs. Has a more superficial relationship with the patient	0.719			
3	Able to work with a broad spectrum of patient/client types vs. Able to work with only a narrow range of patient/client types	0.718			
4	Works effectively in a team vs. Works more effectively alone	0.632			
5	Has a health education role vs. Role is unrelated to health education	0.602			
6	Has the ability to refer a patient to another professional vs. Works with the patient within his or her own professional field of knowledge	0.546			
7	Requires a high level of technical skill vs. Requires a high level of intellectual skill	0.506			
8	Possesses good interpersonal skills with an individual patient vs. Demonstrates good interpersonal skills within a group situation	0.491			
9	Role is clear and transparent to other professionals vs. Uncertainty among other professionals about what the role involves		0.857		
10	Has a broad range of life experience vs. Has little practical life experience		0.835		
11	Seeks out a high degree of involvement with the patient vs. Maintains a low degree of involvement with the patient		0.703		
12	Exudes a high degree of professionalism vs. Does not appear to consider his or her professional image		0.689		
13	Cares for the patient’s general wellbeing vs. Cares for the patient only in relation to his or her specific professional context		0.497		
14	Demonstrates a sense of humour when undertaking his or her role vs. Demonstrates a serious attitude when undertaking his or her role			0.684	
15	Medical focus of the work vs. Social focus of the work			0.612	
16	Has an objective, medical perspective vs. Has a subjective, social perspective			0.529	
17	Has a specific role that involves little collaboration with others vs. Engages in considerable collaboration with others				0.718
18	Works autonomously vs. Works with direction or supervision by another professional				0.657

**Table 3:** Mean scores of factors related to medical students’ attitudes of the role of the nurse.

Factors	Mean Score/SD	LT	EN	*p*
Breadth of Professional Outlook	4.375 (3.375–5.375)	3.875	4.898	<0.001
Projected Professional Image	3.45 (2.5–4.8)	2.8	4.29	<0.001
Possesses Skills for a Wide Professional Scope	3.45 (2.5–4.8; 3.81)	5.0	5.19	0.656
Degree of Professional Interdependence	7.5 (6–8.5)	7.5	6.79	<0.001

## Data Availability

The datasets used and/or analysed during the current study are available from the corresponding author on reasonable request.
